# Retraction: A multifaceted and feasible prognostic model of amino acid metabolism-related genes in the immune response and tumor microenvironment of head and neck squamous cell carcinomas

**DOI:** 10.3389/fonc.2026.1931874

**Published:** 2026-07-20

**Authors:** Frontiers Editorial Office

**Affiliations:** Frontiers Media SA, Lausanne, Switzerland

The Journal retracts the 2022 article cited above.

Following publication, concerns were raised regarding the integrity of the images in the published figures, specifically in **Figure 11**. The authors failed to provide a satisfactory explanation during the investigation, which was conducted in accordance with Frontiers’ policies.

The retraction was approved by the Chief Executive Editor at Frontiers. The authors were informed of the retraction and had an opportunity to respond. This communication has been recorded by the publisher.

Frontiers would like to thank the users on PubPeer for bringing the published article to our attention.

